# Biotransformed soybean cream as a new nutraceutical for skin care: collagen stimulation *in vitro* and *ex vivo*

**DOI:** 10.1590/1414-431X2023e12781

**Published:** 2023-10-20

**Authors:** B.A. Leite, P.H.A. Bezerra, B. Stocco, N. Abichabki, L.N. Andrade, M.J.V. Fonseca, M.R. Torqueti

**Affiliations:** 1 Laboratório de Citologia Clínica, Departamento de Análises Clínicas, Toxicologia e Ciência Alimentar, Faculdade de Ciências Farmacêuticas de Ribeirão Preto, Universidade de São Paulo, Ribeirão Preto, SP, Brasil; 2 Laboratório de Pesquisa em Resistência e Virulência Bacteriana, Faculdade de Ciências Farmacêuticas de Ribeirão Preto, Universidade de São Paulo, Ribeirão Preto, SP, Brasil; 3 Laboratório de Controle de Qualidade, Faculdade de Ciências Farmacêuticas de Ribeirão Preto, Universidade de São Paulo, Ribeirão Preto, SP, Brasil

**Keywords:** Aging, Fibroblasts, Regeneration, Skin Aging, Isoflavones, Menopause

## Abstract

Treatments that attenuate the effects of hypoestrogenism in menopausal women have been gaining visibility. This study investigated the skin response to a phytoestrogen-enriched cosmetic formulation created by incorporating a biotransformed soybean extract (BE) into a cream-like matrix. Collagen-I expression was analyzed both *in vitro* (fibroblast cells) and *ex vivo* (skin explants). The results revealed an increased amount of collagen-I both in fibroblasts and human skin when treated with BE and BE-incorporated cream. Also, this collagen-I overexpression was inhibited by PHTPP, indicating a dependence on estrogen hormone receptor beta (ERβ) signaling. Moreover, BE was not harmful to skin microbiota, showing a promising nutricosmetic potential. Thus, this work presented a fully functional cream-like formulation that was shown to be safe and effectively increase collagen-I levels both *in vitro* and *ex vivo*.

## Introduction

New technologies in cosmetology are being developed to obtain bioproducts capable of improving the quality of human skin, not only improving the visual aspect, but also allowing greater control of biological homeostasis. The skin is composed of a dense mesh of collagen and elastic fibers (extracellular matrix) produced by cutaneous fibroblasts, which guarantee physical consistency, texture, and elasticity (1). Binding of stimulators such as estrogen to estrogen receptors (ERs) activates fibroblasts and results in higher concentrations of secreted components (collagen and elastin fibers), revealing a robust involvement of ERs in the maintenance of skin homeostasis (2).

Collagen is the most abundant structural protein in the human body, comprising various tissues, such as skin (3). Its biological importance is attributed to skin homeostasis, in which types I and III collagen account for up to 90% of this organ (4). Disturbances in the production and decreased levels of this protein occur naturally in menopausal women and are related to the deterioration of the extracellular matrix and estrogen deficiency, leading to fragile tissue (3,5). In fact, studies have demonstrated that estrogens have beneficial and protective effects on skin physiology (6) by promoting acceleration of the healing process and providing protection against photoaging (7).

Isoflavones have shown positive effects on menopause-related skin alterations, such as appearance and composition, by stimulating synthesis and decreasing degradation of dermal collagen (3,5). Phytoestrogens, mainly in the form of isoflavones, are originated from secondary metabolism of plants and have a chemical structure similar to 17β-estradiol (8). Compared with 17β-estradiol, phytoestrogens have a low affinity for ERα and a high affinity for estrogen hormone receptor beta (ERβ), which is more widely distributed in the skin (9).

The bioavailability of aglycone isoflavones, the most active form (8,9), is crucial for their binding to ERs and their estrogenic action. The conversion of glycosylated isoflavones to aglycones can occur naturally *in vivo* or *in vitro* by β-glycosidase enzymes. The effects on skin depend upon the absorption of isoflavones (both cutaneous and oral), their bioavailability in aglycone conformation, and their binding to appropriate ERs in sufficient concentration to stimulate their action (10). The aglycones genistein and daidzein, present mostly in soybean, have been identified as valuable biomolecules in skin care, being able to increase both hyaluronic acid production as collagen I and III levels (3,11).

The conversion of soybean's glycosylated isoflavones to aglycones can be conducted *in vitro* by β-glucosidase enzymes of microorganisms, especially *Aspergillus awamori*, through a fermentation process (12,13). Since the collagen-stimulating activity of biotransformed soybean extracts has been previously shown by our research group (14), the present work investigated a cosmetic formulation for this product, determining its estrogenic activity, collagen stimulation, and preliminary characteristics.

## Material and Methods

### Biotransformation of soybean extracts and isoflavones quantification

The enzyme filtrate was obtained from the fungus *Aspergillus awamori* (CCT5935) and used for the biotransformation of macerated and defatted soybeans (Kodilar, NaturalLife^®^; lot 231-4; Brazil), as previously described (15). For the negative control (non-biotransformed soybean extract - NBE), all the procedures used to obtain the BE were followed, except for the addition of the enzyme filtrate. For the positive control, the commercial soybean extract (CE) containing 41.06% of isoflavones was purchased from Purifarma (Brazil).

The extracts were characterized by measuring the amounts of genistein and daidzein by high-performance liquid chromatography (HPLC) performed on Shimadzu equipment (Japan) equipped with an LC-10AT solvent pump unit and SPD-10a UV-visible detector. Soybean extracts were resuspended in 80% methanol up to 5 mg/mL for BE and NBE and 0.015 mg/mL for the commercial extract. Then, the separation of isoflavones was performed in a reversed-phase column (BDS Hypersil C18, Thermo Scientific, Brazil). Elution was performed in a mobile phase gradient composed of 1% acetic acid in acetonitrile (solvent A) and 1% acetic acid in water (solvent B). After injecting a 20-μL sample, solvent A was increased from 0 to 35% in 90 min at a flow rate of 1 mL/min and UV detection at 280 nm.

Standard solutions of daidzein and genistein, both commercially available (Sigma^®^, USA), were injected in triplicate at increasing concentrations (0.50, 1.25, 2.50, 5.00, 7.50, and 9.00 μg/mL in 80% methanol), generating a linear graph that correlated the concentration of these compounds with peak areas. The identification of daidzein and genistein peaks in soybean extracts was accomplished by comparing the retention time of the extracts' chromatograms with commercial standards, and quantification was made by comparing the chromatogram areas of the BE and NBE peaks and commercial extract to the linear graphs of the standards.

### Antibacterial activity

The antibacterial activity of the BE was investigated against bacterial species commonly found in the human skin microbiome and against bacterial pathogens. Assays were performed by the reference broth microdilution method using the Cation-Adjusted Mueller Hinton II Broth (CAMHB) (BBL™, Becton Dickinson, Brazil) and 96-well polystyrene non-treated microplates with round bottoms. *Staphylococcus aureus* ATCC 29213, *Staphylococcus epidermidis* ATCC 14990, *Staphylococcus saprophyticus* ATCC 15305, *Rhodococcus equi* ATCC 6939, *Enterococcus faecalis* ATCC 29212, *Escherichia coli* ATCC 25922, *Pseudomonas aeruginosa* ATCC 27853, *Acinetobacter baumannii* ATCC 19606, *Klebsiella pneumoniae* ATCC 13883, *Stenotrophomonas maltophilia* ATCC 13637, and *Enterobacter cloacae* ATCC 14087 were evaluated. The assays were performed according to the European Committee on Antimicrobial Susceptibility Testing (16). Polymyxin B and linezolid were used as antibiotic controls in the experiments against Gram-negative and Gram-positive bacteria, respectively. An aqueous solution of resazurin sodium salt (Sigma-Aldrich) was used to assess the metabolic activity and proliferation of bacterial cells, visually determined by bioreduction of the dye (blue) converted into resorufin (pink) in the presence of viable bacteria (17).

### Cell culture

The primary human fibroblast cell line HDFa (human dermal fibroblasts adult) was purchased from Life Technologies (Invitrogen-Cat. No. C0135C, USA), grown in 75-cm^2^ culture flasks in 106 medium (Invitrogen-Cat. No. M106-500) supplemented with low serum growth supplement (LSGS Kit) (Invitrogen-Cat. No. S-003-K), and kept at 5% CO_2_ and 37°C. The evaluation of cytotoxicity in the cellular model had been carried out previously by our research group (14), so no new tests were required for the present study, ensuring safety at the cellular level for the application of this extract in human skin.

#### Treatments

HDFa cells were plated with a culture medium containing 10% fetal bovine serum for 24 h. After this period, the medium was replaced by RPMI medium with 10% charcoal-stripped fetal bovine serum, with BE and NBE at 1.33 μg/mL, which corresponds to 2.27 ng/mL of genistein.

#### Analysis of collagen and estrogen receptors by immunofluorescence

HDFa cells were plated onto a 24-well plate (5×10^4^ cells/well) on coverslips (KNITTEL) for 24 h. After the 24 h treatment, cells were washed, fixed with 2% formaldehyde, and permeabilized for 10 min with 0.3% Triton X-100. The choice of extract concentrations used in the following assays was based on previously performed studies (14), in which the bioavailability of soy extract, daidzein, and genistein was evaluated in cytotoxicity tests with concentrations of 500, 100, 50, 10, 5, 1, 0.5, and 0.1 µg/mL. This allowed extraordinarily high concentrations of soy extract in the blood of animals after oral ingestion of 3 mg of soy extract or isoflavone. The incorporation of 3% soy extract ensured that the level of isoflavones retained in the skin were below the mentioned concentrations.

Sites free of formaldehyde were blocked with 0.25 M glycine in PBS 1X for 5 min. After, the coverslips were blocked for 45 min with 1% BSA (A7906, Sigma) + 5 μg/mL IgG rabbit antibody. Coverslips were incubated with primary anti-collagen type I antibody (Chondrex - 7086, USA) for 1 h at room temperature and subsequently incubated with secondary antibody conjugated to Alexa 488 anti-Rat (A21206, Molecular Probes, USA) 1:350, and DAPI (4',6-diamidino-2-phenylindole) 1:125 for 1 h. The slide was mounted and read using a Leica Microsystems microscope (Leica DM 1000 Led, Germany). Fluorescence intensity was then analyzed. For the investigation of the estrogen role in the production of type I collagen, PHTPP (4-[2-phenyl-5,7-bis (trifluoromethyl) pyrazole [1,5-a]-pyrimidin-3-yl] phenol) highly selective β-blocker was used.

#### Analysis of collagen and estrogen receptors by western blotting

HDFa cells were plated onto 6-well plates (1×10^6^ cells/well) and treated. Cells were trypsinized, centrifuged at 400 *g* for 5 min at 25°C, and lysed with phosphoprotein lysis buffer (20 mM Tris HCl pH7.5, 150 mM NaCl, 1 mM EDTA (salt), and 1% v/v Igepal CA 630) with 1% protease inhibitor and 10% phosphatase inhibitor to obtain the protein extract. Protein quantification of extract was performed by the Pierce™ BCA Protein Assay Kit (Thermo Fisher, USA) based on the absorbance of the solution.

The protein extract (25 µg) was resuspended in sample buffer (100 mMol Tris-HCL, 5% ß-mercaptoethanol, 4% sodium dodecyl sulfate, and 20% glycerol), incubated for 5 min to 100°C and centrifuged at 400 *g* for 5 min at 4°C, and then subjected to gel electrophoresis in 12% polyacrylamide. The samples were then electroblotted onto 0.22-μm nitrocellulose membranes (GE Healthcare, Brazil) and incubated with the primary antibody (1:1,000) anti-procollagen I (Abcam antibodies, ab34710/Lot: GR159090-1, USA) overnight. Membranes were incubated with anti-rabbit secondary antibody (1:10,000) (Jackson Immuno Research, Code: 711-035-152/Lot: 104907, USA), and proteins of interest were visualized by chemiluminescence using the ECL system (GE Healthcare) and ChemiDoc photo documenter (Biorad ChemiDoc XRS + System with Image Lab™ Software, USA). Primary antibody (1:5,000) anti-tubulin α/ß (Cell Signaling Technology, Code: 2148S/Lot: 0005, USA) was used as a protein loading control and the quantification was made with the ImageJ^®^ software (NIH, USA).

### Development and preliminary stability study of a cosmetic formulation containing soybean extracts

After the quantification of the isoflavones daidzein and genistein, a cream-like formulation was prepared to act as a vehicle for soybean extracts (20% TP^®^ stearic acid, 0.02% propylparaben, 0.18% methylparaben, 10% propylene glycol, 1% triethanolamine, 3% soybean extracts, 65.8% deionized water), based on stability results, organoleptic characteristics, pH, temperature, centrifugation, and water loss.

The preliminary stability study was carried out under extreme temperature conditions, with cycles of 24 h at 40±2°C and 24 h at 4±2°C for 12 days. The analysis of the organoleptic and physicochemical characteristics (pH value, centrifugation, and water loss) was always made in newly prepared formulations (control) and at the end of each cycle. Five measurements were made during the 12 days of testing, as previously described (18).

#### Centrifugation test and organoleptic characteristics

The centrifugation test was performed by submitting 2 g of each sample to centrifugation at 1660 *g* for 30 min in a centrifuge at 25°C (Eppendorf 581OR, Brazil). The acceptability criterion was the non-occurrence of phase separation (14).

The samples were visually analyzed for appearance, color, odor, homogeneity, and phase separation, as previously described (18).

#### Determination of pH value

pH was evaluated was by direct determination in 10% (w/v) aqueous solutions of samples using the Digimed DM20 pH-meter (BioVera, Brazil) (18).

#### Water loss

The formulations were monitored for water loss, which was evaluated by weighing aliquots of the formulations and calculating the percent loss of the aqueous fraction.

### Permeation and retention of daidzein and genistein in BE formulations

#### Skin acquisition

The skin from a pig's ear was obtained commercially from a certified slaughterhouse. The skin was cleaned with forceps and a scalpel and stored at -80°C for up to 3 months. At the time of use, the skin was thawed and the fatty tissue was removed with the aid of scissors, isolating the dermis and epidermis.

#### Ex vivo retention and permeation study

This assay was conducted using Franz cells (PermeGear, USA) with a 12-mL receptor solution compartment and a diffusion area of 1.77 cm^2^. The receptor medium consisted of a 150 mM phosphate buffer (pH 7.2) maintained at 37°C under constant stirring at 300 rpm on a magnetic bar, as previously described (14).

The pig ear skin was placed on top of the recipient cell, with the stratum corneum facing the donor compartment and the dermis facing the recipient compartment of the diffusion cell. The skins were treated with 200 mg of the formulations incorporated with 3% BE and NBE, or non-incorporated formulations (placebo), or commercial extract. After 12 h, the skin was removed from the Franz cell compartment, cut into small pieces, and transferred to a conical tube, where 2.5 mL of 80% methanol was added. The skin was then subjected to sonication for 30 min and centrifugation (5000 *g*, 20 min, 4°C), and the supernatant was analyzed by HPLC.

After 12 h of Franz cell operation, the isoflavones present in the recipient liquid were extracted with dichloromethane. Then, the extracted liquid was dried with compressed air, resuspended in 200 µL of 80% methanol, and analyzed by HPLC.

### Analysis of collagen in human skin explants

#### Obtaining, establishing, maintaining, and cultivating organotypic skin explants

This study was approved by the Research Ethics Committee (CEP) of the Faculty of Pharmaceutical Sciences of Ribeirão Preto, University of São Paulo (FCFRP-USP), Brazil (CEP/FCFRP No. 017/2021). Consent was provided by patients who donated skin removed during abdominoplasty. The inclusion criteria for this project were women without hormone replacement therapy, over fifty years of age, non-smokers, and non-users of medication, for at least one month before the skin removal surgery.

The superficial subcutaneous fat was removed from the skin, and then the skin was left overnight in PBS 1X with penicillin (500 U/mL), streptomycin (500 μg/mL), fungizone (25 μg/mL), and gentamicin (250 μg/mL). Any remaining layer of hypodermis was removed, and the material was cut to create a 2-cm^2^ fragment. The skin was cultured in a 1:1 Dulbecco's modified Eagle medium without phenol red with Ham's F-12, (Thermo Fisher Scientific Inc., Cat. No. A4192001) supplemented with 10% fetal bovine serum, 1% l-glutamine, 5 μg/mL insulin, and 10-11 triiodothyronine, pyruvate (1 mM), glutamine (8 mM), penicillin (100 U/mL), streptomycin (100 μg/mL), fungizone (2.5 μg/mL), and gentamicin (50 μg/mL). The dermis was submerged in the culture medium using grids, while the epidermis was kept in contact with the air.

#### Skin treatment

The cream formulation described above was used as a vehicle for the incorporation of 3% BE and 3% NBE. In addition, the cream was used without the addition of extracts as a placebo (vehicle control). As a negative control of the study, skin fragments without cream were used.

The cream formulations were applied evenly to the skin at 2 mg per cm^2^. Each of the proposed conditions were used in triplicate and analyzed at three different times (0, 15, and 30 days). The culture medium and the cream was reapplied every 48 h to the materials.

The tissue fragments were cultured for 30 days, allowing sufficient time for stimulation of collagen-secreting cells and their production. The period of 15 days served as an intermediate phase of the investigation.

After the exposure period, one skin sample was kept in liquid nitrogen for antioxidant and total protein assays, while another fraction was left in 10% formalin with sodium phosphate buffer for 4 h and then transferred to a 70% ethanol solution for histological analysis of the collagen content.

### Obtaining skin extracts

Skin samples were thawed and minced in phosphate buffer (50 mM, pH 7.4) containing EDTA (1 mM), PMSF (1 mM), pepstatin (1 μg/mL), and leupeptin (1 μg/mL) at 200 mg of skin to 2 mL of buffer. Then, the material was homogenized in Turrax^®^, and the suspension was centrifuged at 9.5 *g* for 30 min at 5°C.

#### Ferric reducing antioxidant power (FRAP) in skin

To determine the antioxidant activity through iron reduction, the ability of the antioxidants (reducing agents) in the sample to reduce the Fe^3+^/tripyridyltriazine (TPTZ) complex to form Fe^2+^ in an acidic medium (pH 3.6) was measured at the maximum absorption of 593 nm. The FRAP reagent was prepared at the time of analysis by mixing acetate buffer (300 mM, pH 3.6), TPTZ solution (10 mM TPTZ in 40 mM HCl), and FeCl_3_ (20 mM) in aqueous solution in a 10:1:1 ratio. A 30-μL aliquot of the skin extract solution was added to 150 μL of the FRAP reagent and incubated at 37°C in a water bath for 30 min. Then, the absorbances were measured and the spectrophotometer was blanked with the FRAP solution. The calibration curve was obtained using the Trolox standard curve.

#### Determination of total protein

The quantification of total proteins was determined using the Thermo Scientific™ Pierce™ BCA Protein Assay Kit (Catalog number: 23225), according to the manufacturer’s instructions.

#### Collagen identification and quantification

Collagen identification and quantification were determined by staining assays using picrosirius-hematoxylin/eosin. First, the slides containing the skin sections were fixed with toluene and stained with 0.1% picrosirius for 1 h. Then, the slides were washed and stained with hematoxylin and eosin. The collagen and morphology of the samples were determined by polarized light microscopy using the auto-measure module of the Zeiss Axiovision imaging system (Germany). Collagen identification and quantification were determined in 6 different fields of the epidermis of each section studied with ImageJ^®^ software.

### Statistical analysis

Data were analyzed using GraphPad Prism 6.0^®^ software (GraphPad Software, USA) and are reported as means±SE. Two groups were compared using the Student's *t*-test, while three or more groups were compared two by two using one-way ANOVA together with Tukey *post hoc* analysis. Statistical significance was indicated by P<0.05. At least three independent biological replicates were carried out.

## Results

### BE had greater amounts of isoflavones

The levels of daidzein and genistein in BE, NBE, and commercial extract were quantified by HPLC. The commercial standards of daidzein and genistein generated a linear graph that correlated the concentration of these compounds with the peak areas (Supplementary Figure S1) and was later used to identify isoflavones (Supplementary Figures S2, S3, and S4). It was observed that the BE had a higher amount of daidzein and genistein than NBE (an increase of 35.71 and 20.34%, respectively). However, daidzein levels in the BE and NBE were significantly lower than the commercial extract (Table 1).

**Table 1:** Daidzein and genistein concentrations in soybean extracts.

Soybean extract	Daidzein (mg/g)	Genistein (mg/g)
NBE	0.81±0.02^a^	1.41±0.02^a^
BE	1.26±0.02^b^	1.77±0.01^b^
Commercial	8.44±0.02^c^	0.14±0.01^c^

Data are reported as means±SE of three independent experiments, performed in triplicate. P<0.05, different superscript letters in the same column indicate a statistically significant difference from the other groups (ANOVA and Tukey tests).

### BE had no antibacterial activity

**Figure 1:**
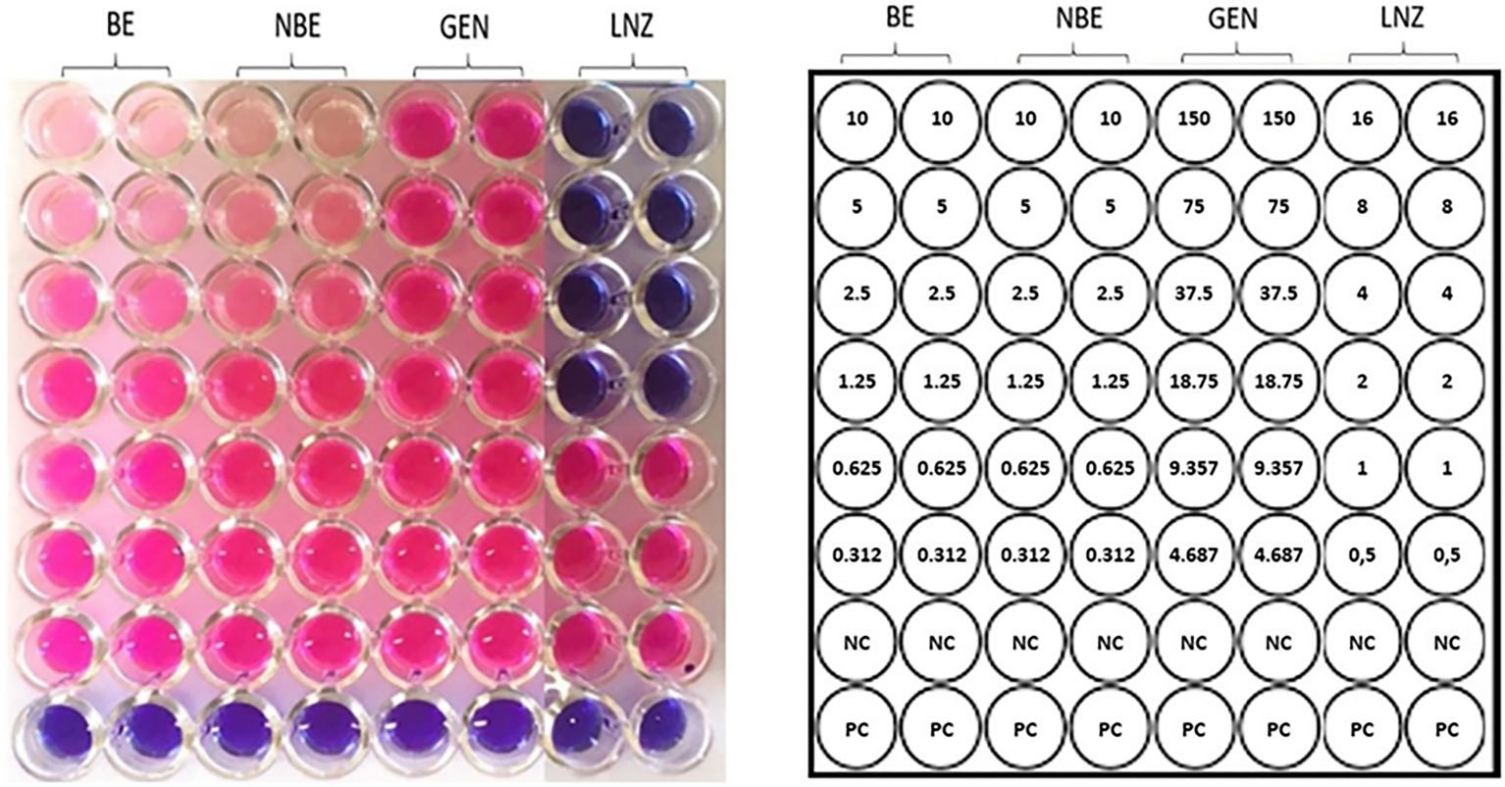
*Staphylococcus epidermidis* treated in duplicate with BE (biotransformed transgenic soybean extract), NBE (non-biotransformed soybean extract), GEN (genistein), and LNZ (linezolid). Blue wells represent bacterial growth inhibition and pink wells indicate bacterial growth. The numbers in the left panel indicate the concentration of the compounds in each well. The negative control (NC) was a natural bacterial growth, shown in pink color. The positive control (PC) was a sterilized culture medium, with no growth, shown in blue color.

To verify if soybean extracts were harmful to skin microbiota bacteria, antibacterial activity was evaluated by the broth microdilution method, with the determination of minimum inhibitory concentration (MIC). Bacterial species usually found in the skin microbiota, such as *Staphylococcus aureus*, *S. epidermidis*, *S. saprophyticus*, *Escherichia coli*, *Pseudomonas aeruginosa*, *Klebsiella pneumoniae*, *Enterobacter cloacae*, and *Enterococcus faecalis*, as well as some opportunistic pathogens with different morphological characteristics, such as *Rhodococcus equi* and *Stenotrophomonas maltophilia*, were included. Results did not show BE MIC for concentrations up to 150 µg/mL against all strains tested (Supplementary Figures S5-S8). For example, no antibacterial activity was found for *S. epidermidis* (Figure 1) at the tested concentrations, showing no damage to the common skin microbiota.

### Increased collagen-I expression in HDFa cells

Cell and tissue viability assay was previously performed by our research group (14,15) following already established and validated processes.

**Figure 2:**
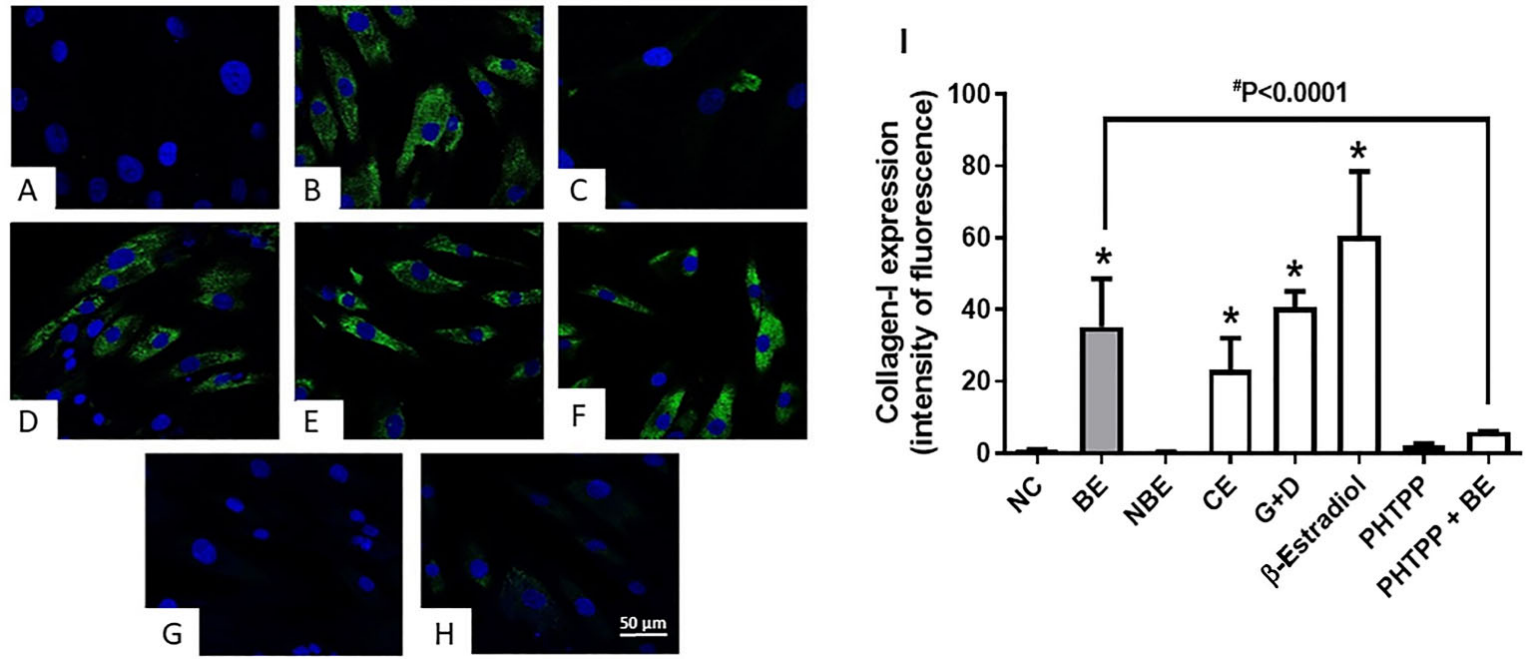
Fluorescence microscopy of primary human dermal fibroblasts adult (HDFa) labeled with anti-procollagen type I antibody (green) and DAPI (blue) after 24 h exposure to treatments. **A**, Cells with culture medium only (NC, negative control); **B**, Cells treated with biotransformed soy extract (BE, 1.33 μg/mL); **C**, Cells treated with non-biotransformed soy extract (NBE, 1.33 μg/mL); **D**, Cells treated with commercial soy extract (CE, 0.023 μg/mL); **E**, Cells treated with genistein + daidzein (G, 1.90 and D, 2.27 ng/mL); **F**, Cells treated with β-estradiol (50 ng/mL); **G**, Cells treated with PHTPP (1 μM); **H**, Cells treated with PHTPP (1 μM) + BE (1.33 μg/mL). Magnification 40×, scale bar 50 μm. **I**, Quantification of collagen-I expression in HDFa. Data are reported as means±SE of three independent experiments. *P<0.05 *vs* NC, (ANOVA and Tukey *post hoc* test).

**Figure 3:**
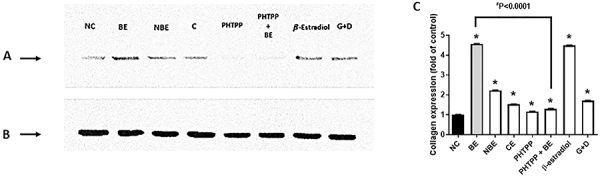
Collagen-I expression in human dermal fibroblasts adult (HDFa) cells after 24 h of treatment. **A**, Expression of type 1 collagen protein. **B**, Expression of ß-tubulin protein. **C**, Quantification of collagen expression (fold of control). Data are reported as means±SE of three independent experiments. *P<0.05 *vs* NC (ANOVA and Tukey post-test). Negative control (NC, medium only); biotransformed soy extract (BE, 1.33 µg/mL); non-biotransformed soy extract (NBE, 1.33 µg/mL); commercial soy extract (CE, 0.023 μg/mL); PHTPP (1 μM); PHTPP (1 μM) + BE (1.33 μg/mL); β-estradiol (50 ng/mL); genistein + daidzen (G+D, 1.90 + 2.27 ng/mL).

Immunofluorescence revealed that BE significantly increased collagen-I expression in relation to the negative control. Interestingly, when BE was combined with the ER-β inhibitor PHTPP, its ability to stimulate collagen-I expression was significantly decreased (Figure 2). Western blot analysis for collagen-I expression confirmed these immunofluorescence findings (Figure 3 and Figure S9).

The green fluorescence in the cytoplasm of HDFa cells (Alexa 488) under the different treatments and controls is shown in Figure 2A-H. The quantification of the fluorescent intensity was performed using Leica Microsystems microscope (Leica DM 1000 Led, LAS X^®^) and is shown in Figure 2 (panel I).

### Parameters of the BE-enriched cream

**Figure 4:**
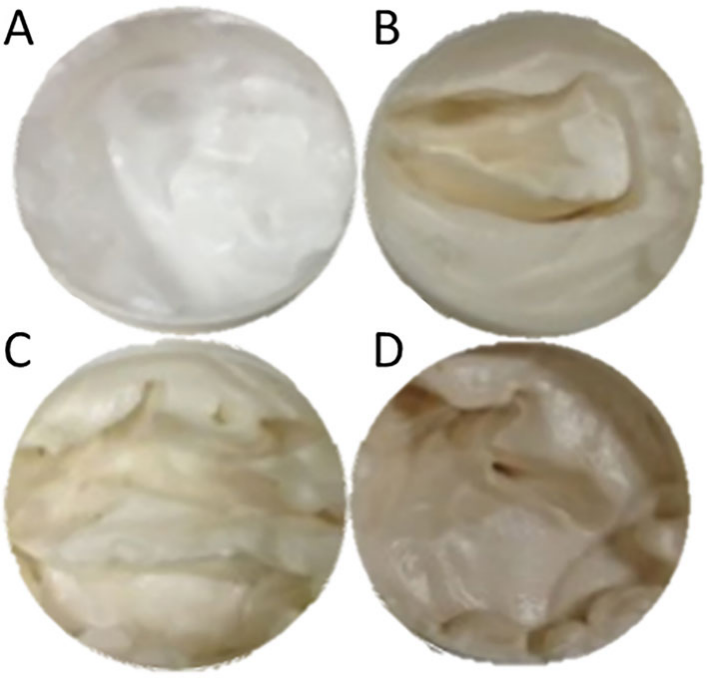
Macroscopic appearance of cream formulations with or without the incorporation of extracts, **A**, Placebo (formulation only); **B**, Formulation incorporated with non-biotransformed soybean extract; **C**, Formulation incorporated with biotransformed soybean extract; **D**, Formulation incorporated with commercial soybean extract.

**Figure 5:**
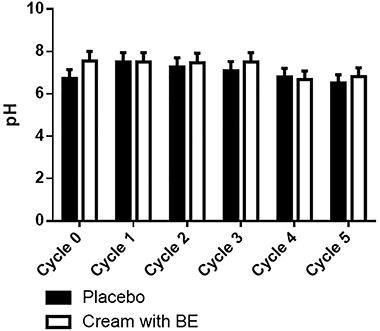
pH values of cream formulations incorporated or not (placebo) with the biotransformed soy extract (BE). Data are reported as means±SE (ANOVA); n=6 measurements, performed on newly prepared formulations (Cycle 0) and at the end of each cycle.

**Figure 6:**
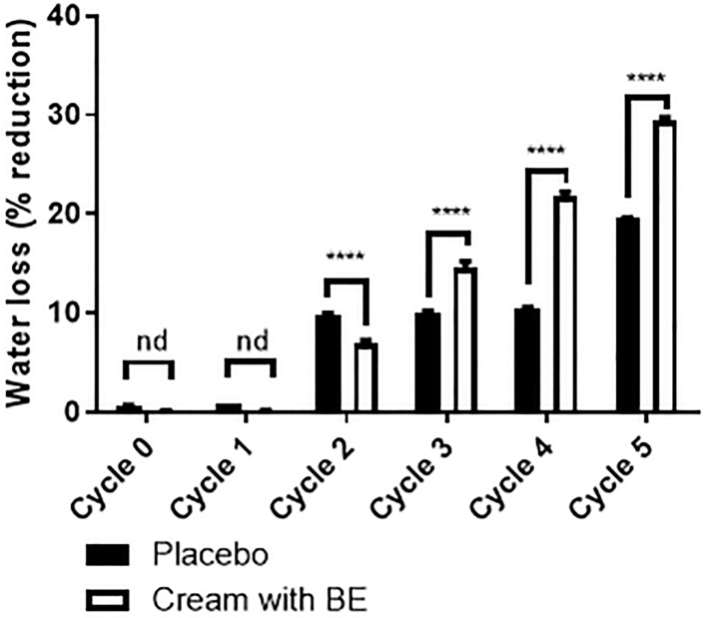
Water loss of cream formulations incorporated or not (placebo) with the biotransformed soy extract (BE). Data are reported as means±SE; n=6 measurements, performed on newly prepared formulations (Cycle 0) and at the end of each cycle. ****P=0.006 (ANOVA and Tukey *post hoc* test). nd: not detected.

Neither the BE-enriched nor the other formulations presented alterations in organoleptic features during the 12 days of study or after centrifugation cycles (Figure 4). After the stability test, the BE-enriched formulation showed pH values between 4.2 and 7.55 (Figure 5) and a difference in water loss (Figure 6).

### BE-enriched cream allowed retention of isoflavones and their permeation into the pig's ear skin

**Figure 7:**
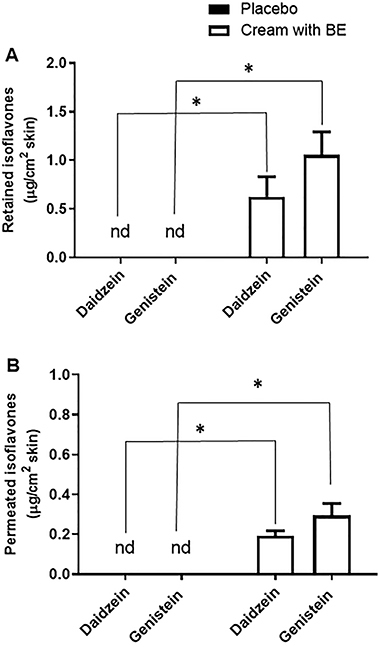
Retention (**A**) and permeation (**B**) in the Franz cells of isoflavones daidzein and genistein after the pig's ear skin treatment with cream formulations incorporated or not (placebo) with the biotransformed soybean extract (BE). Data are reported as means±SE of six independent experiments. *P=0.05 *vs* placebo (ANOVA and *t*-test). nd: not detected.

The retention test showed that the amount of daidzein in the pig's ear skin after 12 h of treatment with the cream formulation was 0.6266 µg/cm^2^, while the amount of genistein retained in the skin was 1.0555 µg/cm^2^ (Figure 7A). Moreover, the cream formulation enabled the penetration of isoflavones into the pig's skin, resulting in a permeation of 0.1921 µg/cm^2^ of daidzein and 0.2954 µg/cm^2^ of genistein (Figure 7B). The retention and permeation comparisons were between placebo (cream) and BE cream.

### BE-enriched cream increased the expression of collagen-I in human skin explants

**Figure 8:**
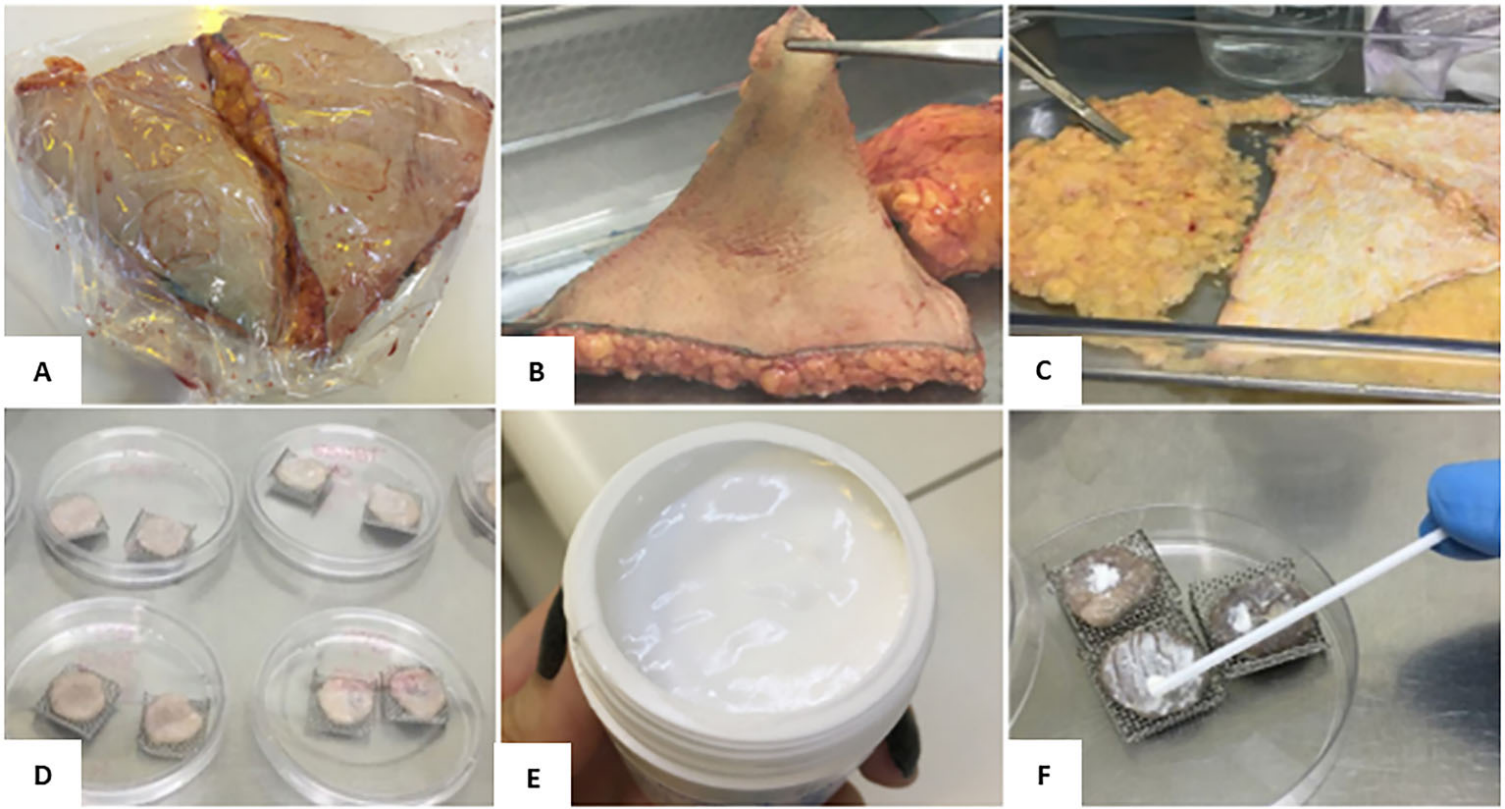
Processing of human skin fragments. The skin fragment (**A** and **B)** was separated from the adipose tissue (**C**), and the explants were cut (2 cm^2^) and cultivated on stainless steel grids inside 90×15-mm plates in contact with the culture medium (**D**), kept at 37°C and 5% CO_2_, so that it was possible to add the formulation under study (**E** and **F**).

The analysis of type I collagen production in human skin was assessed by the human organotypic skin explant culture model (hOSEC) analysis, which is a promising alternative method to mimic the cutaneous distribution profile of drugs and cosmetics. Figure 8 describes the processing steps of the human skin, which was submitted to cleaning, conditioning in the culture system, and application of the formulations onto the skin explants.

**Figure 9:**
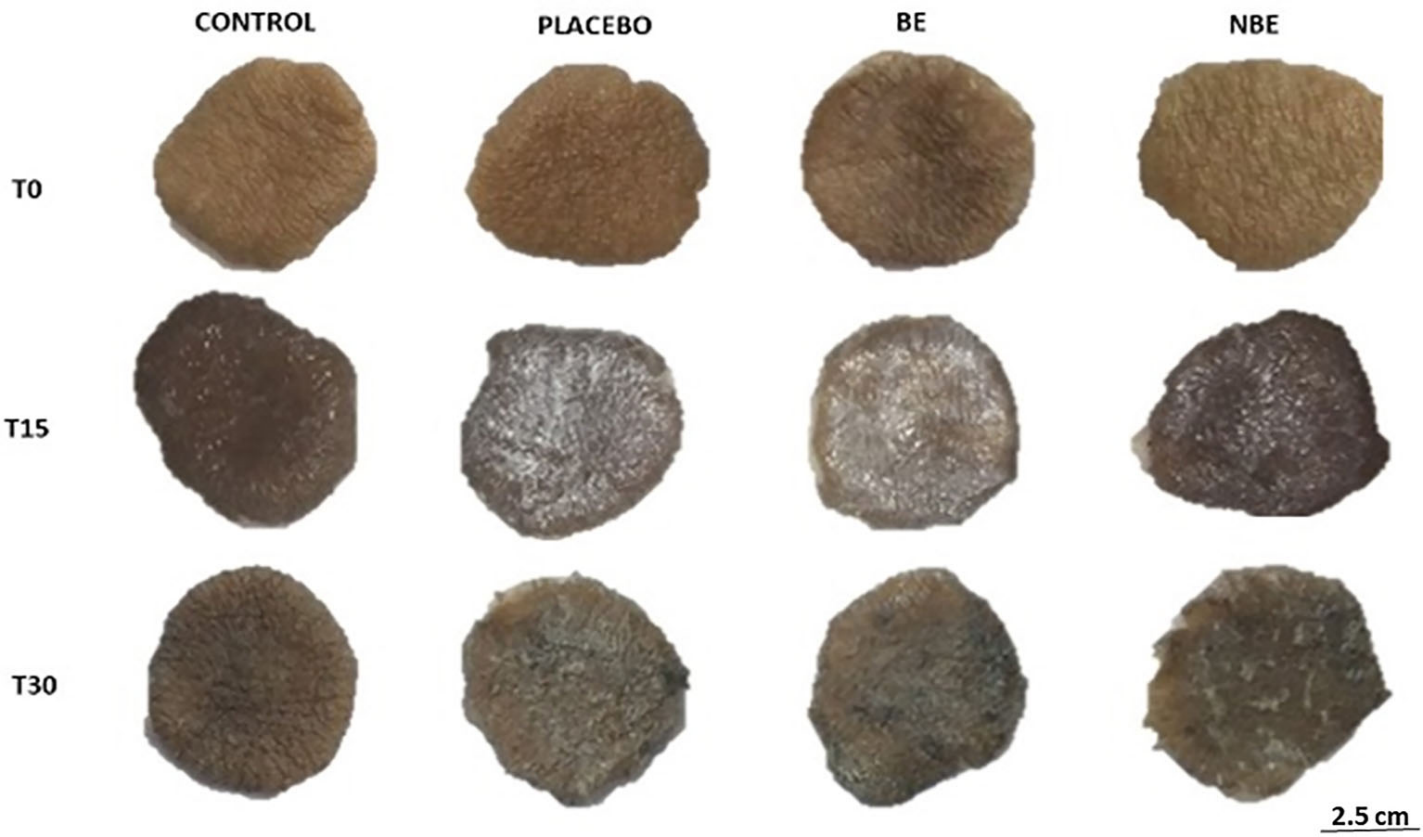
Fragments of human skin at 0 days (T0), 15 days (T15), and 30 days (T30) of cultivation. Images are shown under the same conditions of analysis, and show skin fragments without cream application (CONTROL), with cream application (PLACEBO), with application of biotransformed soy extract incorporated into the cream (BE), and with application of non-biotransformed soy extract incorporated into the cream (NBE). Scale bar 2.5 cm.

The human skin fragments incubated in the culture medium at 37°C in 5% CO_2_ with humidified air showed visual color changes after 15 and 30 days of culture in relation to day 0 (Figure 9).

**Figure 10:**
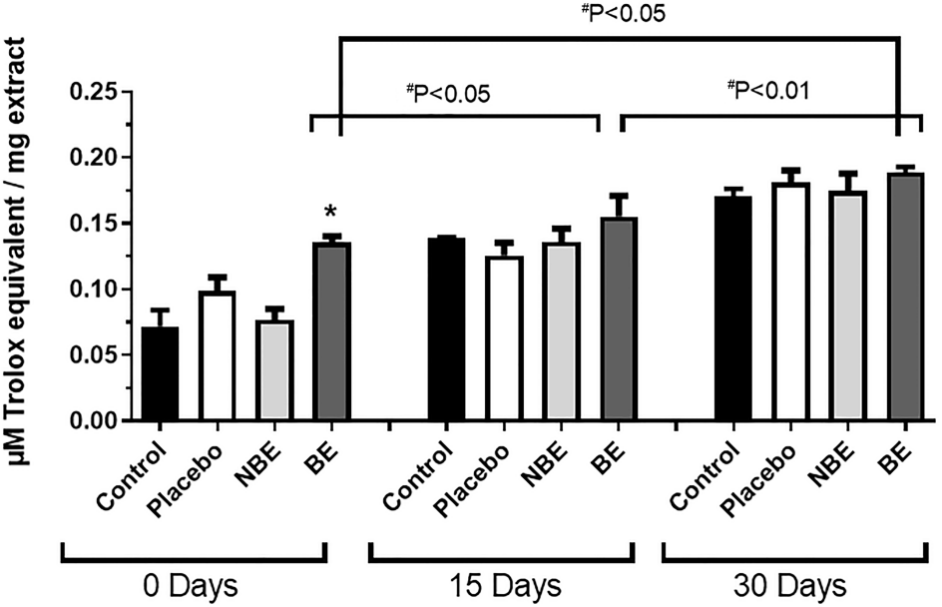
Ferric reducing ability of plasma (FRAP) in human skin extracts. Results are reported as means±SE of three independent experiments, performed in triplicate with human skin extracts treated for 0, 15, and 30 days with biotransformed soy extract (BE), non-biotransformed soy extract (NBE), and placebo, in addition to skin without any application as a negative control. *P<0.05 compared to control (ANOVA and Tukey *post hoc* test).

The FRAP assay showed a significant increase in antioxidant activity in the skin explants treated with BE-enriched cream compared to the placebo, and the increase was time-dependent (Figure 10).

**Figure 11:**
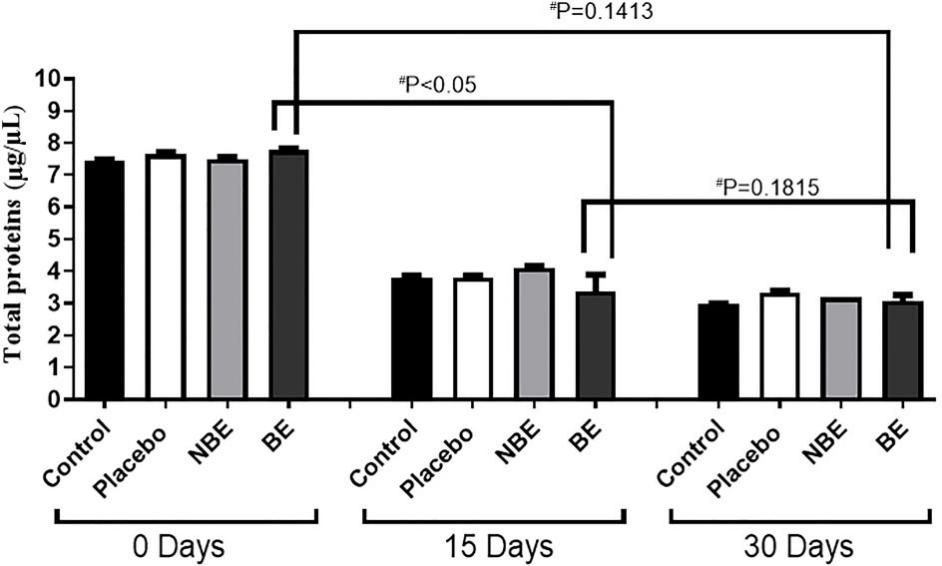
Quantification of total protein in human skin extracts. Results are reported as means±SE of three independent experiments, performed in triplicate with human skin extracts treated for 0, 15, and 30 days with biotransformed soy extract (BE), non-biotransformed soy extract (NBE), and placebo, in addition to skin without any application as a negative control (ANOVA and Tukey *post hoc* test).

The total protein of treated skin explants was quantified by BCA assay, and it was not different between treatments. Moreover, a significant decrease in total protein was noted after 15 days, independent of the treatment (Figure 11).

**Figure 12:**
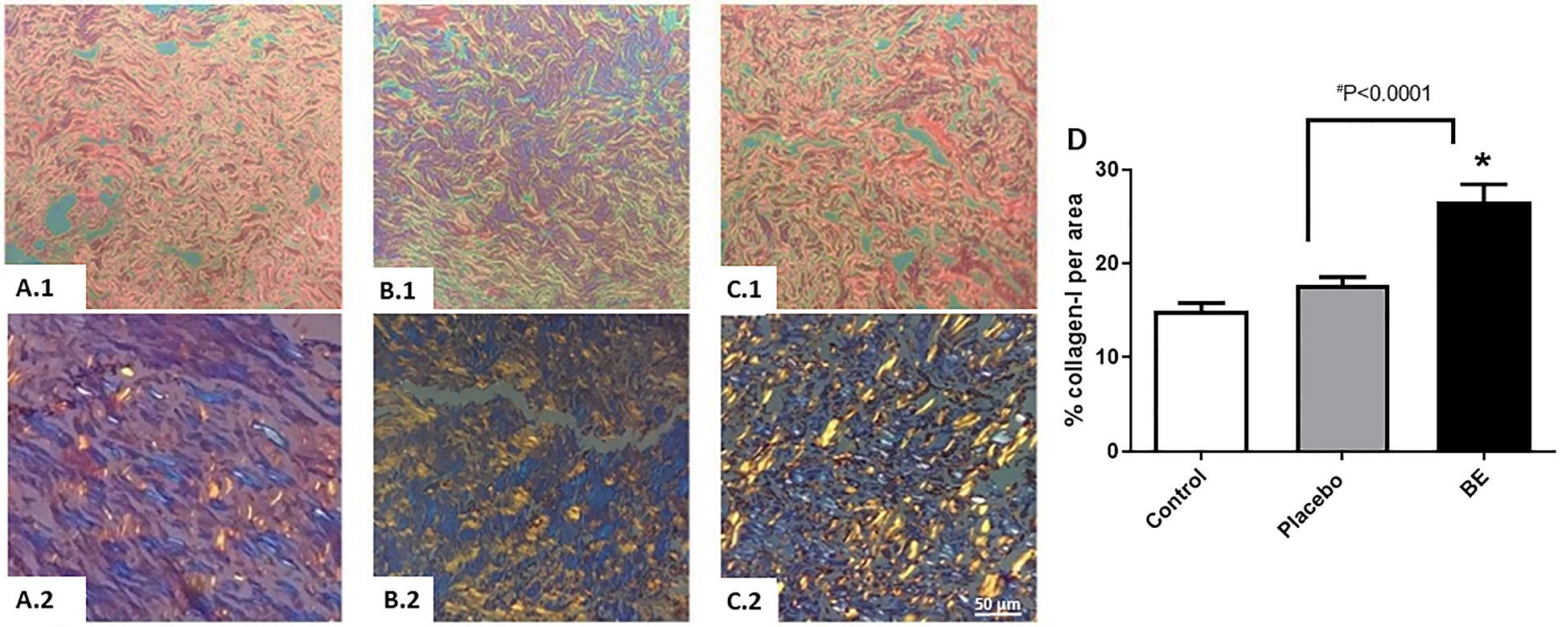
Micrographs in polarized light (**A.2**, **B.2**, **C.2**) and without polarized light (**A.1**, **B.1**, **C.1**) of human skin samples after 30 days of treatments, showing in orange type I collagen (picrosirius red). **A**, human skin explant; **B**, human skin explant treated with placebo; **C**, human skin explant treated with biotransformed soybean extract (BE) cream formulation. Magnification 40×, scale bar 50 μm. **D**, Quantification of collagen-I. Data are reported as means±SE of three independent experiments. *P<0.05 *vs* negative control (ANOVA and Tukey *post hoc* test).

Type I collagen (Figure 12) was identified and quantified by picrosirius-hematoxylin staining in human skin explants, showing an increase in skins treated with the BE-enriched cream for 30 days compared to the negative control (an increase of 43.89%) and compared to the placebo (an increase of 33.63%).

## Discussion

The development of both health and cosmetic products should meet the demand for new technologies that provide products with fewer side effects obtained from safe raw materials that are easily obtained. In the present work, biotransformation of soybean extracts generated an increase in the concentration of isoflavones daidzein and genistein, corroborating previous studies (14). In fact, soybean extracts have gained great importance in the area of skin health due to the marked presence of secondary metabolites with medicinal effects, such as isoflavones, that might help in the combat of skin damage effects of menopause (3,11). The isoflavones genistein and daidzein have been identified as important biomolecules that can exert beneficial responses in the side effects of hormone decline, especially those related to estrogen depletion during menopause (19).

Lower estrogen levels in menopause can decrease collagen expression (20). In the analysis of collagen by western blotting and immunofluorescence, the 1.33 µg/mL BE concentration was optimal for type I collagen stimulation. This collagen-I production seems to be linked to the ERβ interaction as previously reported (21), since when combined with PHTPP, the BE loses its ability to increase collagen expression. Our results also showed that the BE did not cause damage to the beneficial bacteria that are part of the natural microbiota of human skin, ensuring safety for the use of the product in the industry (22).

In order to show the benefits of a potential product, the present work has developed a cosmetic formulation with these extracts. The cream formulation showed good stability, with minimum alterations of the original form over time, which is essential for the acceptance of a product by the market (23), and with pH values between 4.2 and 7.55. Since the pH of the skin surface is around 4.5 to 7.2, it is desirable that the pH of products for topical use is within this range of values (24). The cream formulation allowed the permeation and retention of both daidzein and genistein into the skin after 12 h. In fact, the presence of carboxylic acid at the stearic acid end in isoflavones has already shown the ability to facilitate the penetration of compounds, since they have a close similarity to the constituents of the skin (25).

Human skin explants showed visual changes in color after 15- and 30-day cultivation compared to 0-day fragments regardless of treatment. The literature shows that skin fragments cultured for 75 days under the same conditions showed thinning, but the dermo-epidermal junction was unchanged and there was proliferation of cells expressing keratinocytes (26). Moreover, the FRAP study showed an increase in antioxidant activity in samples of skins treated with BE. Several studies have shown that increased FRAP activity directly reflects an increase in antioxidant capacity in oxidative skin injury (27). In this case, the greater the capacity and quantity of the natural antioxidants of the skin samples to interact with this ion, the greater the conversion data in relation to the equivalent μM trolox/mg skin extract. These results can be explained by the application of the extract itself, which contained isoflavones (genistein and daidzein) in a significantly higher concentration in relation to soybean extracts that did not undergo the biotransformation process, acting as antioxidant agents. The soybean isoflavone extract acts as an anti-photoaging agent in skin care, showing protective effects in UVB-induced oxidative stress and keratinocyte death (28).

The dermis is essentially composed of the extracellular matrix (ECM) and fibroblasts, and during aging or tissue degradation, significant changes occur, such that collagen, the main component of the ECM, becomes fragmented and its total concentration decreases (26). However, total protein level was not significantly different for the BE group, decreasing over time as in the other treatment groups. Indeed, the literature shows that the degradation mechanism when the material is out of its homeostasis, as in tissue culture, is explained by the activity of matrix metalloproteinases and the impairment of the transforming growth factor beta signaling induced by reactive oxygen species (28).

It has been previously shown that nanofibrous dressings developed from plant sources with soybean protein hydrolysate are able to improve wound healing with tissue reconstitution and collagen stimulation in the skin (29). The present work corroborates this by presenting a BE cream formulation that increased the content of type I collagen in skin explants, which is probably not related to the antioxidant capacity of the collagen extract but may be related to the interaction of one of the extract components with ERβ in the skin. Using the standard controls of purified genistein and daidzein, it can be concluded that collagen production may be influenced by the isoflavones in BE. However, it should also be taken into account that despite the strong evidence that isoflavones, especially genistein, are responsible for the increased collagen expression, it is believed that genistein may not be solely responsible for this effect, since chromatographic analyses show that the extract has several other components.

Finally, it was demonstrated that the transgenic soybean extract biotransformed by the fungus *Aspergillus awamori* at 1.33 μg/mL promoted the expression of type I collagen in primary human fibroblasts and in human skin explants compared to the non-biotransformed extract. The present work also showed a fully functional cream formulation that enhanced collagen-I expression and had high nutricosmetic potential, as it not only had a valuable effect on the skin but also did not interfere with the natural microbiota.

Thus, future complementary skin tests are suggested for a better understanding of the process and improvement of the developed technique.

## Supplementary material

Click to view [pdf].
